# Clinical Evaluation Before MRI Referral: Frequency and Association with Diagnostic Yield

**DOI:** 10.3390/tomography12060082

**Published:** 2026-06-01

**Authors:** Zahra H. M. Alquraish, Yuki Arita, Thomas C. Kwee

**Affiliations:** 1 Medical Imaging Center, Department of Radiology, University Medical Center Groningen, University of Groningen, 9700 RB Groningen, The Netherlands; 2 Department of Radiology and Biomedical Imaging, Division of Molecular Imaging and Therapeutics, University of California, San Francisco, CA 94143-0628, USA; yukiarita1113@gmail.com; 3 Department of Radiology, Memorial Sloan Kettering Cancer Center, New York, NY 10065, USA

**Keywords:** magnetic resonance imaging, MRI referral, history taking, physical examination, clinical reasoning, diagnostic yield

## Abstract

Purpose: To evaluate how often history taking and physical examination are omitted before MRI referral and whether their omission is associated with clinical reasoning quality and MRI diagnostic yield. Materials and Methods: In this prospective study, adults undergoing MRI at a tertiary academic hospital were surveyed before imaging to determine whether the referring clinician had taken their history and performed a physical examination. Multivariable regression was used to assess determinants of omission and associations with clinical reasoning quality (defined as agreement between the suspected diagnosis and MRI findings) and MRI positivity (defined as findings relevant to the indication). Results: Among 275 patients (median age 61 years; 50.0% male), history taking was omitted in 18.2% of cases and physical examination was omitted in 70.9%. History taking was less likely during surveillance than during new/first visits (odds ratio (OR) 0.140, *p* < 0.001) and more likely when MRI was requested by residents rather than medical specialists (OR 4.645, *p* = 0.018). Physical examination was more likely when MRI was requested by residents (OR 3.174, *p* = 0.007) or nurse specialists/physician assistants (OR 3.145, *p* = 0.033), and less likely during follow-up visits (OR 0.183, *p* < 0.001) and surveillance visits (OR 0.061, *p* < 0.001). Omission of physical examination was not associated with clinical reasoning quality (*p* = 0.370). Neither omission of history taking nor omission of physical examination was associated with MRI positivity (*p* = 0.430 and *p* = 0.286, respectively). Conclusions: History taking and physical examination were often omitted before MRI referral. Although no statistically significant association was observed between omission of bedside assessment and clinical reasoning quality or MRI positivity, reduced bedside assessment may limit the clinical context informing referral and interpretation.

## Simple Summary

Magnetic resonance imaging (MRI) is widely used, but it should ideally be guided by a careful patient history and physical examination. In this prospective study of 275 adults referred for MRI, history taking was omitted in 18.2% of cases, and physical examination in 70.9%. These omissions were not statistically significantly linked to poorer agreement between the suspected diagnosis and imaging findings, or to whether the scan showed relevant abnormalities. However, missing bedside assessment may reduce the clinical context available to radiologists. These findings support better referral information, renewed attention to bedside skills, and future studies on when pre-imaging assessment is most important.

## 1. Introduction

MRI is an important diagnostic tool, but its use is not always justified and may contribute to avoidable healthcare costs [1]. Imaging overuse has become an increasingly recognized problem, and the appropriateness of MRI requests may vary according to the clinical indication. An unnecessary MRI may also lead to incidental findings unrelated to the presenting complaint, which can prompt further imaging, specialist referral, or invasive testing. In addition, avoidable imaging adds to the workload of radiologists, which has already increased substantially in recent years [2]. Furthermore, MRI is frequently performed with gadolinium-based contrast agents, which may cause immediate hypersensitivity reactions [3], can be retained in the body, and contribute to environmental contamination [4,5]. Therefore, more judicious use of MRI is warranted.

Ideally, MRI referrals are guided by bedside clinical reasoning: medical history and physical examination help narrow the differential diagnosis, determine whether MRI is appropriate, and improve the expected yield of imaging [6]. Clear referral information, including relevant history, examination findings, and a suspected or differential diagnosis, also helps the radiologist understand the clinical question and interpret imaging findings in the context of the patient’s presentation [7,8,9,10,11,12]. However, the importance of history taking and physical examination may vary depending on the purpose of the MRI examination, such as evaluation of a new complaint compared with follow-up of a known disease. At the same time, bedside assessment may be used less consistently in routine practice, with clinicians increasingly relying on imaging [12,13,14], alongside reduced emphasis on physical examination in training and clinical workflows [15,16,17,18,19]. It remains unclear how often history taking and physical examination are omitted before MRI referral in routine practice. Better insight into this may help improve referral quality and reduce low-value imaging.

The purpose of this study is therefore to examine how often history taking and physical examination are omitted prior to MRI referral and whether this affects diagnostic yield.

## 2. Methods

### 2.1. Study Design

This prospective study was conducted at <BLINDED>, a tertiary academic referral hospital in the Northern Netherlands. The protocol was approved by the local institutional review board, and all participants provided written informed consent. Between 5 January and 11 February 2026, patients in the MRI waiting area were approached shortly before their examination by a research fellow, <BLINDED>, contingent on the fellow’s availability, resulting in a pragmatic convenience sample. Adults (≥18 years) undergoing MRI for clinical indications were eligible. Exclusion criteria were inability/unwillingness to consent, critical illness, sedation, legal incapacity, inability to communicate in Dutch or English, and MRI performed solely for research purposes.

### 2.2. Data Collection

Patient characteristics recorded included age, sex, referring department, and the type of healthcare professional who requested the MRI examination (resident, medical specialist, nurse specialist/other). The clinical indications for MRI were categorized as (i) evaluation of a new complaint or symptom, (ii) follow-up imaging (defined as imaging performed after an earlier test or established diagnosis to assess treatment response, progression, or resolution), or (iii) surveillance imaging (defined as scheduled imaging in asymptomatic patients aimed at detecting recurrence or new pathology). The anatomical region examined (e.g., brain, spine, extremities, or abdomen) was also recorded for each case. Patients were approached by the research fellow in the MRI waiting area, either before or after their scan, depending on patient flow and the fellow’s availability. They completed a paper-based questionnaire, which included the informed consent statement and is provided in Appendix A. The research fellow discussed the questions and response options verbally with each patient to ensure understanding. The questionnaire asked whether their referring clinician had performed medical history taking and/or a physical examination before referral (“yes,” “no,” or “unsure”); responses were documented by the research fellow, and patients who answered “unsure” were excluded from further analyses.

### 2.3. MRI Yield and Clinical Reasoning

MRI results were categorized as positive (i.e., findings relevant to the clinical indication), negative (i.e., no relevant abnormalities, no evidence of progression, and no new clinically significant changes compared with prior imaging, if available), or indeterminate (i.e., findings that were nonspecific, equivocal, or technically limited, precluding a definitive diagnosis or confirmation or exclusion of the suspected condition). Clinical reasoning quality was quantified as a percentage concordance score (0–100%), reflecting the agreement between the suspected diagnosis or differential diagnoses stated on the MRI request form and the MRI findings, in line with previous work [6,9]. For example, if three conditions were listed (e.g., cerebral ischemia, vasculitis, and a space-occupying lesion) but only cerebral ischemia was confirmed on brain MRI, the assigned score was 33%. Complete agreement between the suspected and confirmed diagnoses was scored as 100%, whereas completely discordant findings were scored as 0%. If no suspected diagnosis or differential diagnosis was provided, or if the MRI result was classified as indeterminate, no clinical reasoning score was calculated.

### 2.4. Statistical Analysis

The proportions of patients who reported that they had not undergone history taking or physical examination by their referring physician before MRI were calculated, along with 95% confidence intervals (CIs). Multivariable logistic regression analyses were performed to identify factors associated with omissions of history taking and omissions of physical examinations, including patient age, sex, main indication, referring specialty group, healthcare professional who requested the MRI, and MRI scan site/anatomical region of interest. Subsequently, multivariable linear regression was performed in patients with an available clinical reasoning score to assess the association between the omission of a physical examination before MRI referral and clinical reasoning quality, adjusted for main indication, referring specialty group, healthcare professional who requested the MRI, and MRI scan site/anatomical region of interest. Patients without a documented differential diagnosis and those with indeterminate MRI findings were excluded from this analysis. History taking was not included in the final model because only a small number of patients in this subset had no documented history before MRI referral. Finally, multivariable logistic regression was used to evaluate the association between omission of history taking or physical examination prior to MRI referral and MRI positivity, adjusting for the same variables as described above, after exclusion of scans with indeterminate results. Statistical analyses were conducted using IBM SPSS Statistics version 28. *p*-values < 0.05 were considered statistically significant.

## 3. Results

### 3.1. Patients

**Figure 1:**
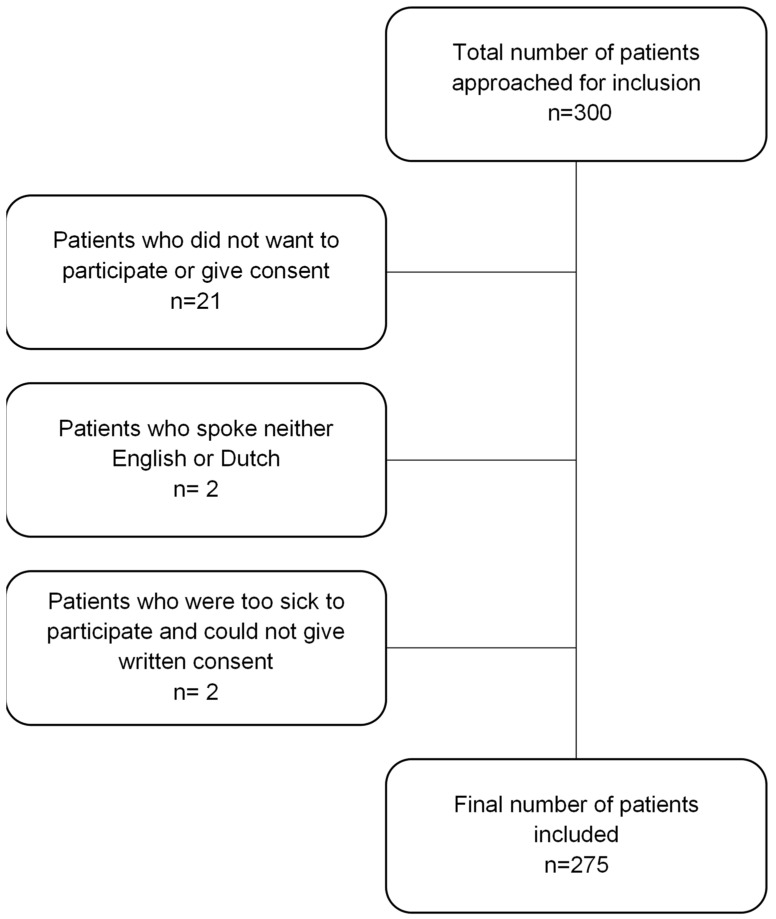
Patient inclusion flowchart.

During the study period, 300 patients were approached for participation, of whom 275 were included in the final cohort (Figure 1). Table 1 presents the characteristics of the 275 patients included in the study. The median age was 61 years (range, 18–87 years), and 140 participants (50.9%) were male, and 135 (49.1%) were female. Most referrals originated from non-surgical specialties (69.5%), while 30.5% came from surgical specialties. The most common referring departments were gastroenterology (16%), neurology (12.7%), and radiation therapy (12.0%). Most MRI scans were requested by medical specialists (69.8%), followed by residents (20.4%) and nurse specialists/physician assistants (9.8%). The main indication for MRI was follow-up (46.9%), followed by new or first-visit evaluations (26.9%) and surveillance (26.2%). The most frequently examined anatomical region was the brain/head (39.6%), followed by the abdomen (36.0%) and chest (10.9%).

### 3.2. Omission of History Taking: Frequency and Determinants

History taking was omitted in 50 of 275 patients (18.2%), while 225 patients (81.8%) had reported that history taking had been performed before the MRI referral. In multivariable logistic regression, visit type was significantly associated with overall history taking (*p* < 0.001). Compared with new/first visits, surveillance visits were significantly less likely to include history taking (OR 0.140, 95% CI 0.047–0.413; *p* < 0.001), whereas follow-up visits were not significantly different (OR 0.511, 95% CI 0.170–1.537; *p* = 0.232). The referring clinician position was also significantly associated with overall history taking (*p* = 0.031). Referrals made by residents were more likely to include history taking than referrals made by specialists/consultants (OR 4.645, 95% CI 1.298–16.626; *p* = 0.018), whereas referrals made by nurse specialists/physician assistants were not significantly different (OR 2.680, 95% CI 0.682–10.532; *p* = 0.158). Age, sex, anatomical site, and specialty group were not significantly associated with history taking (Table 2).

### 3.3. Omission of Physical Examination: Frequency and Determinants

Physical examination was omitted in 195 of 275 evaluable patients (70.9%, 95% CI: 65.3 to 76.0%). Physical examination was significantly more likely to be performed when the MRI was requested by a resident (OR 3.174, *p* = 0.007) or a nurse specialist/physician assistant (OR 3.145, *p* = 0.033) compared with a medical specialist. Physical examination was also more often performed before brain MRI (OR 3.622, *p* = 0.004) and in other anatomical regions (OR 3.573, *p* = 0.018) compared with abdominal MRI. Physical examination was significantly less likely to be performed at follow-up visits (OR 0.183, *p* < 0.001) and surveillance visits (OR 0.061, *p* < 0.001) compared with new or first visits. Age, sex, and requesting specialty group were not significantly associated with the performance of a physical examination prior to MRI (Table 3).

### 3.4. Omission of Physical Examination vs. Clinical Reasoning Quality

In 176 patients, no clinical reasoning score could be calculated because the referral form did not contain a documented differential diagnosis, or the MRI result was classified as indeterminate. In the remaining 99 patients, omission of physical examination before MRI referral was not significantly associated with clinical reasoning quality (B = −8.418; *p* = 0.370). Anatomical region was significantly associated with overall clinical reasoning quality (*p* = 0.034), and brain MRI was associated with lower clinical reasoning scores compared with other MRI sites (B = −28.552; *p* = 0.015). None of the other variables included were significantly associated with clinical reasoning quality.

### 3.5. Omission of Physical Examination vs. Diagnostic MRI Yield

Neither omission of physical examination nor omission of history taking prior to MRI was significantly associated with MRI positivity (physical examination: OR 0.657, 95% CI 0.304–1.420; *p* = 0.286; history taking: OR 1.389, 95% CI 0.614–3.145; *p* = 0.430). Visit type was significantly associated with overall MRI positivity (*p* = 0.005). Compared with MRI scans requested at a new/first visit, surveillance MRI was significantly less likely to yield a positive result (OR 0.211, 95% CI 0.083–0.539; *p* = 0.001), whereas follow-up MRI was not significantly different (OR 0.543, 95% CI 0.255–1.155; *p* = 0.113). No significant associations were observed for age, sex, referring specialty, anatomical site, or referring clinician position (Table 4).

## 4. Discussion

The results of our study show a striking phenomenon in pre-MRI clinical assessment: history taking was omitted in 18.2% of cases, while physical examination was omitted in 70.9% of cases. History taking was less often performed during surveillance visits. This likely reflects the follow-up nature of surveillance care, in which the underlying condition is already established, and imaging is requested within a routine clinical pathway. In this setting, clinicians may be less inclined to repeat history taking and physical examination before referral. This is further reflected by the lower rate of positive MRI scans in the surveillance setting, which is consistent with surveillance imaging being more protocol-driven than prompted by new symptoms. Residents were more likely to take a history, and physical examination was also more often performed when an MRI was requested by a resident, a nurse specialist, or a physician assistant. This reflects the idea that closer supervision during training reinforces the habit of taking a history and performing a physical examination as part of routine clinical decision-making. By contrast, specialists and consultants may see patients later in the referral pathway and rely on referral letters from other physicians who have already performed the history and physical examination. They may also work under greater time pressure and with more competing tasks, leaving less opportunity for bedside assessment. Physical examination was performed more often before brain MRI, possibly because neurological symptoms can often be localized clinically, making bedside assessment particularly useful for guiding the differential diagnosis prior to imaging. Yet, brain MRI was associated with lower clinical reasoning scores than other MRI sites. One explanation could be that patients referred for brain imaging often present with broad or nonspecific symptoms, while functional disorders may also be encountered in neurological practice, making it more difficult to formulate a suspected diagnosis that closely matches the eventual MRI findings.

Several studies have raised the concern that bedside clinical skills are receiving less emphasis in modern practice. A retrospective analysis of junior doctors’ admission notes from 1975 to 2010 found that physical examination quality declined over time, reflected in a less extensive assessment and less frequent reporting of signs such as palpable liver, palpable spleen, cardiac murmur, and apex beat location and character [8]. The authors attributed this decline to increasing reliance on diagnostic technology, possibly together with heavy workloads [8]. Similar concerns have also been reported at the trainee level. A large UK study of the Practical Assessment of Clinical Examination Skills (PACES) found that only 49% of 6820 candidates passed on their first attempt, despite specific preparation and direct observation by experienced clinicians beforehand. Some house staff even paid for external physical examination programs to prepare for the exam [15]. This suggests that bedside examination skills may require continued reinforcement beyond formal training [20]. This is also supported by evidence from the 2011 USMLE Step 2 Clinical Skills examination, where 29,442 U.S. medical students performed substantially worse in physical examination than in history taking, with average scores of 59.6% versus 78.1%, respectively [21]. The weakness was not uniform across domains: physical examination scores were lowest for neurological and musculoskeletal encounters, with mean scores of 51.4% and 52.0%, compared with 72.7% for gastrointestinal encounters [13]. These examples suggest that bedside examination deficiencies may be particularly relevant in clinical areas where correct technique and targeted examination are required, such as eliciting reflexes, testing vibratory sensation, or performing focused musculoskeletal assessment [6,22].

Medical education literature has likewise linked the erosion of bedside teaching to greater reliance on diagnostic tests, while traditional clinical skills may be valued less when imaging is expected to provide a rapid answer [15,17,18,19]. The clinical consequences may be important, as inadequate history taking and physical examination have been associated with delayed diagnosis, unnecessary testing, higher costs, and potentially harmful outcomes for patients [15,17,18,22]. This concern is reinforced by evidence that nearly half of diagnostic errors in outpatient care are linked to problems with the physical examination [22]. This may be especially relevant in a clinical environment where residents spend as little as 12% of their time in indirect patient contact and close to 50% in front of the computer [13]. These findings should also be viewed in the context of broader changes in diagnostic practice. Bruls and colleagues suggested that near-unlimited access to advanced imaging may reduce the need for careful clinical selection, effectively replacing clinical reasoning quality with scan quantity [2]. Although bedside assessment itself was not specifically addressed, this supports the idea that increasing reliance on imaging may shift diagnostic decision-making away from clinical evaluation [14]. Against this background, structured requisition frameworks such as the Reason for Exam Imaging Reporting and Data System (RI-RADS), a grading system used to assess the quality and completeness of radiology requests, have been proposed to better align imaging requests with clinical reasoning and improve referral quality [11,18,23]. The importance of referral quality is further supported by studies showing that clinical information can directly influence imaging interpretation [9,10,24]. In a systematic review of 22 observer-performance studies, 15 studies found improved diagnostic performance when clinical history was available, although two more recent studies showed that clinical history increased location sensitivity but also reduced specificity, indicating that clinical information should be relevant and well formulated rather than simply extensive [24]. This is important for MRI referral because the clinical question can guide the radiologist’s search pattern and interpretation. In another article, it was noted that poor-quality referral information may misdirect a goal-directed search, whereas a logically formulated diagnostic question can reduce perceptual and interpretive errors [25].

Our findings have important clinical and practical implications. MRI interpretation depends on adequate clinical context, and without prior history taking or physical examination, MRI may be requested on a less clearly defined clinical basis. Radiologists should therefore remain aware that basic clinical assessment may not always have been performed before imaging. When relevant clinical information is missing from the request, direct communication with the referring clinician may help place MRI findings in the proper context. Our results also suggest that, when bedside assessment is omitted, MRI may take on a larger role in the diagnostic workup. This may add pressure to MRI services, particularly in settings where scan volumes and reporting demands are already high. These findings support greater attention to referral quality, for example, through structured request forms that prompt clinicians to provide key clinical information and a suspected diagnosis. The high rate of omissions of physical examination may also justify renewed attention to bedside skills in clinical training. Future research should examine in which clinical contexts omission of bedside assessment before imaging is acceptable and in which contexts it may compromise the quality of referral and interpretation.

While the present study offers valuable insights, several limitations should be noted. Our assessment of whether history taking and physical examination occurred was based on patient self-report rather than by direct observation or review of clinical encounters. This may have introduced recall bias or misclassification, as patients may not accurately remember or recognize whether a physical examination was performed, potentially affecting the validity of associations involving these variables. Second, this was a single tertiary care academic center study conducted at a tertiary academic hospital, using a convenience sample over a limited study period, which may reduce generalizability to other healthcare settings or populations. Third, clinical reasoning quality could only be calculated when a suspected or differential diagnosis was provided and MRI findings were not indeterminate, which limited the analysis to a smaller subgroup, reduced statistical power, and may have introduced selection bias if patients with calculable scores differed from those without calculable scores. Fourth, clinical reasoning quality was defined by concordance between suspected diagnoses on the MRI request form and MRI findings. Although practical and reproducible, this approach has conceptual limitations, as MRI is not always a definitive gold standard, may oversimplify the complexity of clinical reasoning, and gives equal weight to all listed differential diagnoses without accounting for their order, likelihood, or clinical importance. Therefore, the concordance score should be interpreted as a measure of diagnostic concordance rather than a complete assessment of clinical reasoning quality. Fifth, MRI positivity was defined based on imaging findings relevant to the clinical indication, but this does not fully capture the clinical usefulness of MRI, since negative MRI findings in follow-up or surveillance settings may still be clinically important by confirming stability or absence of progression. In addition, the need for physical examination may vary according to the referring specialty, clinical indication, and purpose of imaging. Sixth, information documented on the MRI request form may not fully reflect the clinician’s actual reasoning or the information considered during clinical decision-making, and this study did not evaluate whether MRI findings changed treatment decisions, management plans, or patient outcomes, which should be addressed in future research.

## 5. Conclusions

History taking and physical examination were often omitted before MRI referral. Although no statistically significant association was observed between omission of bedside assessment and clinical reasoning quality or MRI positivity, reduced bedside assessment may limit the clinical context informing referral and interpretation.

## Figures and Tables

**Table 1:** Characteristics of the 275 included patients.

Characteristics	Summary
Age (median, range)	61, 18–87 years
Sex distribution (no. and %)	- Male 140 (50.9%) - Female 135 (49.1%)
Specialty distribution (no. and %)	- Non-surgical 191 (69.5%) - Surgical 84 (30.5%)
Referring department (no. and %)	- Gastroenterology 44 (16%) - Neurology 35 (12.7%) - Radiation therapy 33 (12.0%) - Cardiology 22 (8.0%) - Otorhinolaryngology 18 (6.5%) - Neurosurgery 14 (5.1%) - Oncology 15 (5.5%) - Gynecology/Obstetrics 10 (3.6%) - Endocrinology 10 (3.6%) - Pulmonology 10 (3.6%) - Hepatobiliary surgery 10 (3.6%) - Orthopedics 8 (2.9%) - Surgical oncology 8 (2.9%) - Urology 6 (2.2%) - Hematology 6 (2.2%) - Geriatrics 5 (1.8%) - Nephrology 3 (1.1%) - mmunology 3 (1.1%) - Ophthalmology 3 (1.1%) - Oral and maxillofacial surgery 3 (1.1%) - Plastic surgery 2 (0.7%) - Trauma surgery 2 (0.7%) - Pediatrics 1 (0.4%) - Vascular surgery 1 (0.4%) - Rheumatology 1 (0.4%) - Dermatology 1 (0.4%)
Healthcare professional who requested the MRI scan (no. and %)	- Medical specialist 192 (69.8%) - Resident 56 (20.4%) - Nurse specialist/Physician assistant 27 (9.8%)
Main indication for MRI (no. and %)	- New/first visit 74 (26.9%) - Follow-up 129 (46.9%) - Surveillance 72 (26.2%)
Anatomical region (no. and %)	- Brain/head 109 (39.6%) - Abdomen 99 (36.0%) - Chest 30 (10.9%) - Extremities 14 (5.1%) - Spine 13 (4.7%) - Pelvis 8 (2.9%) - Multiple regions 2 (0.7%)

**Table 2:** Multivariate logistic regression analysis of factors associated with taking history before MRI.

Variable	Category	Odds Ratio	95% CI	*p*-Value
Patient’s age	NA	0.990	0.971–1.009	0.296
Patient’s gender ^a^	Female	0.683	0.345–1.352	0.274
Requesting medical specialty ^b^	- Surgical	1.234	0.327–4.656	0.757
	- Non-surgical	1.263	0.350–4.556	0.721
Healthcare professional who requested the MRI ^c^	- Resident	4.645	1.298–16.626	0.018
	- Nurse specialist/PA	2.680	0.682–10.532	0.158
Clinical indication for MRI ^d^	- Follow-up	0.511	0.170–1.537	0.232
	- Surveillance	0.140	0.047–0.413	<0.001
Anatomical region of interest ^e^	- Brain/Head	0.675	0.292–1.561	0.358
	- Chest	0.452	0.150–1.367	0.160
	- Others	1.137	0.314–4.121	0.845

Abbreviations: CI: confidence interval. NA: not applicable. Notes: ^a^ Male was used as reference category, ^b^ Neurology was used as reference category, ^c^ Medical specialist was used as reference category, ^d^ New/first visit was used as reference category, ^e^ Abdomen was used as reference category.

**Table 3:** Multivariate logistic regression analysis of factors associated with performing a physical examination before MRI.

Variable	Category	Odds Ratio	95% CI	*p*-Value
Patient’s age	NA	0.989	0.972–1.007	0.231
Patient’s gender ^a^	Female	0.876	0.437–1.754	0.708
Requesting medical specialty ^b^	- Surgical	1.249	0.404–3.865	0.700
	- Non-surgical	0.392	0.128–1.201	0.101
Healthcare professional who requested the MRI ^c^	- Resident	3.174	1.380–7.298	0.007
	- Nurse specialist/Physician assistant	3.145	1.094–9.040	0.033
Clinical indication for MRI ^d^	- Follow-up	0.183	0.085–0.395	<0.001
	- Surveillance	0.061	0.021–0.177	<0.001
Anatomical region of interest ^e^	- Brain/Head	3.622	1.502–8.731	0.004
	- Chest	0.760	0.160–3.601	0.729
	- Others	3.573	1.249–10.225	0.018

Abbreviations: CI: confidence interval. NA: not applicable. Notes: ^a^ Male was used as reference category, ^b^ Neurology was used as reference category, ^c^ Medical specialist was used as reference category, ^d^ New/first visit was used as reference category, ^e^ Abdomen was used as reference category.

**Table 4:** Multivariable binary logistic regression analysis on the association of several factors with a positive MRI result (*n* = 239, excluding 36 cases with indeterminate MRI results).

Variable	Category	Odds Ratio	95% CI	*p*-Value
Patient’s age	NA	1.002	0.987–1.018	0.749
Patient’s gender ^a^	Female	0.990	0.564–1.739	0.973
Requesting medical specialty ^b^	- Surgical	1.177	0.407–3.406	0.764
	- Non-surgical	1.701	0.579–5.000	0.334
Healthcare professional who requested the MRI ^c^	- Resident	1.202	0.579–2.491	0.622
	- Nurse specialist/PA	0.450	0.152–1.332	0.149
Clinical indication for MRI ^d^	- Follow-up	0.543	0.255–1.155	0.113
	- Surveillance	0.211	0.083–0.539	0.001
Anatomical region of interest ^e^	- Brain/Head	0.518	0.248–1.083	0.080
	- Chest	0.546	0.193–1.541	0.253
	- Others	1.268	0.521–3.084	0.601
History taking before the scan ^f^	- Omitted	1.389	0.614–3.145	0.430
Physical examination before the scan ^g^	- Omitted	0.657	0.304–1.420	0.286

Abbreviations: CI: confidence interval. NA: not applicable. Notes: ^a^ Male was used as reference category, ^b^ Neurology was used as reference category, ^c^ Medical specialist was used as reference category, ^d^ New/first visit was used as reference category, ^e^ Abdomen was used as reference category, ^f^ History taking performed used as reference category, ^g^ Physical examination performed used as reference category.

## Data Availability

The datasets generated and/or analyzed during the current study are available from the corresponding author on reasonable request due to patient privacy and ethical restrictions.

## Abbreviations

MRI, magnetic resonance imaging; OR, odds ratio.

## Appendix A. English Translation of the Original Dutch Patient Questionnaire Used to Assess Whether History Taking and Physical Examination Were Performed Prior to MRI Referral

**Verbal information given to the patient by the researcher:** “Dear Mr./Ms. [patient name], we are conducting a study on how often doctors ask patients about their symptoms and perform a physical examination before their scan, and whether this influences the scan result. For this purpose, we would like to ask you three questions. Answering these questions will take no more than one minute of your time. Would you like to participate?”
To be completed by the researcher: Did your doctor ask you further questions about your symptoms, if you had any, before this scan? YES/NO/UNSURE Did your doctor physically examine you before this scan? YES/NO/UNSURE
To be completed by the patient: Do you give us permission to process your age, sex, reason for the scan, and scan result for our study? YES/NO Date: ……………………… Signature: ………………………
